# Low compartment pressure and myoglobin levels in tibial fractures with suspected acute compartment syndrome

**DOI:** 10.1186/s12891-018-2394-y

**Published:** 2019-01-05

**Authors:** Abraham Nilsson, Björn Alkner, Patrick Wetterlöv, Stefan Wetterstad, Lars Palm, Jörg Schilcher

**Affiliations:** 1 Department of Orthopedics and Department of Clinical and Experimental Medicine, Linköping University, Linköping, Sweden; 2 Department of Orthopedics, Regional Hospital Eksjö, Region Jönköping County and Department of Clinical and Experimental Medicine, Linköping University, Linköping, Sweden; 3 Department of Orthopedics, Region Hospital Kalmar, Kalmar, Sweden

**Keywords:** Acute compartment syndrome, Fasciotomy, Myoglobin, Creatine phosphokinase, Tibial fracture

## Abstract

**Background:**

The intense ischemic pain of acute compartment syndrome can be difficult to discriminate from the pain related to an associated fracture. Lacking objective measures, the decision to perform fasciotomy is often only based on clinical findings and performed at a low threshold. Biomarkers of muscle cell damage might help to identify and monitor patients at risk. In patients with fractures, however, markers of muscle cell damage could be elevated because of other reasons associated with the trauma, which would make interpretation difficult. In a review of all patients who underwent emergency fasciotomy in our health care district we aimed to investigate the decision-making process and specifically the use of biomarkers in patients with and without fractures.

**Methods:**

In the southeast health care region of Sweden 79 patients (60 men) with fractures (median age 26 years) and 42 patients (34 men) without associated fractures (median age 44 years) were treated with emergency fasciotomy of the lower leg between 2007 and 2016. Differences in clinical findings, p-myoglobin and p-creatine phosphokinase as well as pressure measurements were investigated.

**Results:**

P-myoglobin was analyzed preoperatively in 20% of all cases and p-creatine phosphokinase in 8%. Preoperative levels of p-myoglobin were lower in patients with fractures (median 1065 μg/L, range 200–3700 μg/L) compared with those without fractures (median 7450 μg/L, range 29–31,000 μg/L), *p* < 0.05. Preoperative intracompartmental pressure was lower in the fracture group (median 45 mmHg, range 25–90 mmHg) compared with those without fractures (median 83 mmHg, range 18–130 mmHg), *p* < 0.05.

**Conclusions:**

Biomarkers are seldom used in the context of acute fasciotomy of the lower leg. Contrary to our expectations, preoperative levels of p-myoglobin and intracompartmental pressures were lower in fracture patients. These findings support differences in the underlying pathomechanism between the groups and indicate that biomarkers of muscle cell necrosis might play a more important role in the diagnosis of acute compartment syndrome than previously thought.

## Background

Acute compartment syndrome of the lower leg is a severe and dreaded complication. Tissue swelling secondary to trauma or ischemia is hampered in the lower leg because of thick fascial layers surrounding the muscle groups. Instead of tissue expansion, the pressure in the muscle compartment increases. As a consequence, oxygen delivery and elimination of metabolic end products are deprived. A vicious circle of increasing pressure and ischemia develops, ultimately leading to muscle cell necrosis*.*

Clinically, acute compartment syndrome is mainly characterized by its intense ischemic pain, tense muscle compartments and neurological deficits. In the situation of a concomitant fracture the pain related to compartment syndrome might be difficult to discriminate from the pain related to the fracture [1]. Because reliable, objective diagnostic criteria of compartment syndrome are lacking and the consequences of an untreated acute compartment syndrome are detrimental, the condition is often treated by surgical decompression with fasciotomy already at a low level of suspicion. This situation is reflected by a large variation in the frequency of emergency fasciotomy in tibial fractures, which is between 2 and 24% depending on the treating surgeon [2]. Fasciotomy is not a harmless procedure. In patients with tibial fracture it is associated with more frequent surgical complications, longer hospital stays, higher costs, and a higher risk of infection and non-union [3–6]. Unnecessary fasciotomies should therefore be avoided.

Intracompartmental pressure measurement is an attempt to introduce objective measures in the decision-making process and observation of patients at high risk [1, 7]. Certain thresholds to perform a fasciotomy have been defined [8]. However, such thresholds are associated with higher fasciotomy rates than with clinical observation alone (18 vs. 4%) [9], suggesting that unnecessary fasciotomies might be performed using this method [10, 11].

Biomarkers indicating muscle cell damage (p-myoglobin and p-creatine phosphokinase) could be used to objectively identify rhabdomyolysis associated with acute compartment syndrome at an early stage [12, 13]. However, in the context of acute compartment syndrome, very little evidence exists to support the use of biomarkers and diagnostic thresholds are lacking. Especially in patients with an underlying fracture in which the source of the pain is difficult to discriminate, an objective measure would be desirable. However, in these patients levels of biomarkers might be elevated because of other trauma-related disorders, such us direct skeletal muscle injury and myocardial injury [13].

This study aimed to describe the decision-making process and specifically the use of biomarkers of muscle cell damage before and after an acute decompressive fasciotomy and to compare patients with underlying fractures to those without fractures.

## Methods

### Patients

We searched the electronic medical and surgical registry for all orthopedic departments in the southeast health care region of Sweden for patients treated with emergency fasciotomy of the lower leg due to acute compartment syndrome between 2007 and 2016 (NOMESCO Classification of Surgical Procedures, code NGM09). In the whole health care district fasciotomy and the preoperative evaluation before a fasciotomy are performed by the orthopedic surgeon. The registries contain personal identification numbers (PINs) of the patients, date of surgery, diagnosis codes, and treatment codes. The validity of the registry is considered high but has never been formally evaluated. Complete linkage between the registries and the medical record is possible through the use of the PIN provided to all Swedish residents. Data from one hospital, covering roughly 5% of the population in the region, could not be obtained. Fasciotomies associated with vascular emergencies were excluded.

Based on the review of the medical notes and radiographs, patients were divided into two groups. One group consisted of patients who had undergone acute fasciotomy and had associated tibial fractures (open and closed injuries). The other group included those without associated fractures (e.g., contusions, crush injuries, peri- or postoperative complications, drug-induced bleedings, and others). The trauma mechanism was classified into three categories (high-energy trauma, low-energy trauma, or no trauma). Fractures were divided after review of individual radiographs into proximal, diaphyseal, and distal tibial fractures (ankle fractures were not included). Proximal fractures were further subclassified according to Schatzker’s classification [14].

P-myoglobin and p-creatine phosphokinase levels were registered pre- and postoperatively in reference to the time point of fasciotomy (hours). Medical notes were reviewed to determine the reasons why biomarkers were analyzed. Intracompartmental pressures were obtained with a handheld pressure measurement device (Stryker Surgical, Kalamazoo, Michigan) or, in four cases, with an intra-arterial blood pressure measurement device.

Perioperative findings for muscle viability were collected from surgical notes based on the 4 Cs (color, consistency, contractility, and capacity to bleed) and methods of wound closure were registered as either primary, secondary, split skin graft, or flaps. The number of amputations of the affected limb was obtained for each group based on medical notes.

### Decision making

The decision-making process before the fasciotomy was obtained by review of medical notes from the different hospitals in the southeast health care region of Sweden. Only those variables clearly mentioned in preoperative and operative notes from the responsible orthopedic surgeons and their teams were regarded to have contributed in the process. Values that were analyzed, though not mentioned in the notes, were not considered.

### Population and data on tibial fractures from national registries

According to registry data from the Swedish National Board of Health and Welfare, 7668 patients at all ages sustained a fracture of the tibia (*International Classification of Diseases*, *10th Revision* [ICD-10], diagnosis codes S82.1x, S82.2x, S82.3x, S82.7x, S82.8x, and S82.9x) in our health care district during the study period. The district comprises one level-1 trauma university hospital, two regional emergency hospitals and three district hospitals with emergency care. At the end of the study period, the health care district counted 1,050,000 inhabitants (Statistics Sweden 2016).

### Statistics

The overall data were examined using descriptive statistics for frequencies and percentages for categorical variables and means, medians and standard deviations and confidence intervals for continuous variables. Comparisons between groups were performed with the chi-square test for frequencies and the Mann-Whitney U test for continuous data. Because of small numbers, no statistical calculations were made for p-creatine phosphokinase. All analyses were performed using IBM SPSS Statistics, Version 25.

## Results

### Patients

**Fig. 1:**
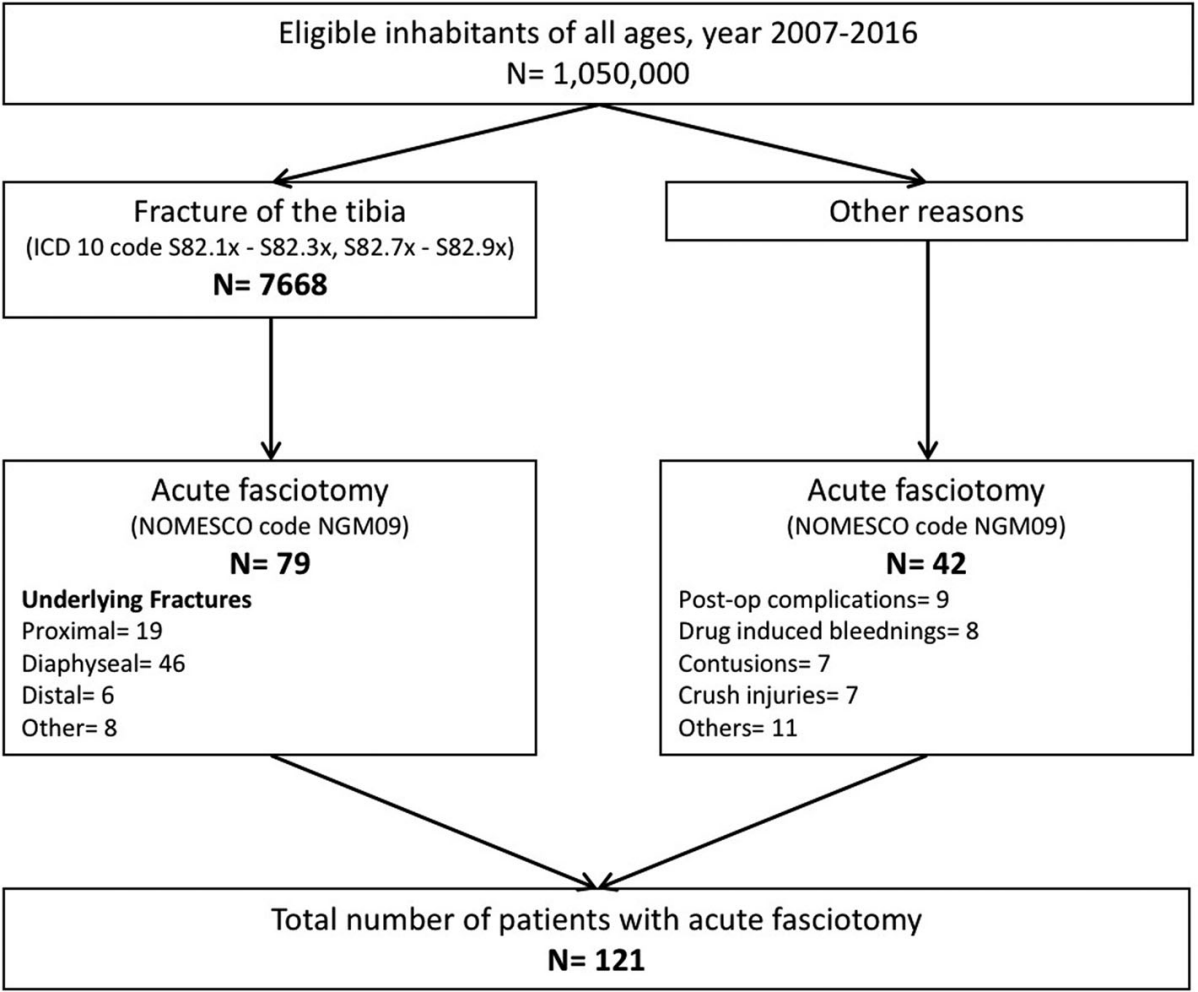
Identification of the two patient groups in the study population

We identified 121 patients, 79 patients (60 men) with fractures (median age 26 years) and 42 patients (34 men) without fractures, who underwent acute fasciotomy of the lower leg during the study period (Fig. 1). Patient characteristics are given in Table 1.

**Table 1:** Patient characteristics

	Age in years median (range)	Males	Trauma mechanism (high- energy trauma/low-energy trauma/no trauma)
All (*n* = 121)	35 (13–80)	94	43/53/25
Fractures (*n* = 79)	26 (13–79)	60	39/40/0
Proximal (*n* = 19)	30 (15–55)	14	10/9/0
Diaphyseal (*n* = 46)	23,5 (13–77)	37	26/20/0
Distal (*n* = 6)	35 (13–79)	3	0/6/0
Other* (*n* = 8)	26,5 (22–67)	6	3/5/0
Non-fractures (*n* = 42)	44 (13–80)	34	4/13/25
Postop complications (*n* = 9)	49 (13–70)	6	0/0/9
Drug-induced bleedings (*n* = 8)	56 (25–80)	7	1/1/6
Contusion (n = 7)	44 (13–64)	7	0/7/0
Crush injuries (*n* = 7)	43 (21–70)	6	2/5/0
Others (*n* = 11)	39 (26–80)	8	1/0/10

*Combined fracture patterns

### Decision making and clinical findings

**Fig. 2:**
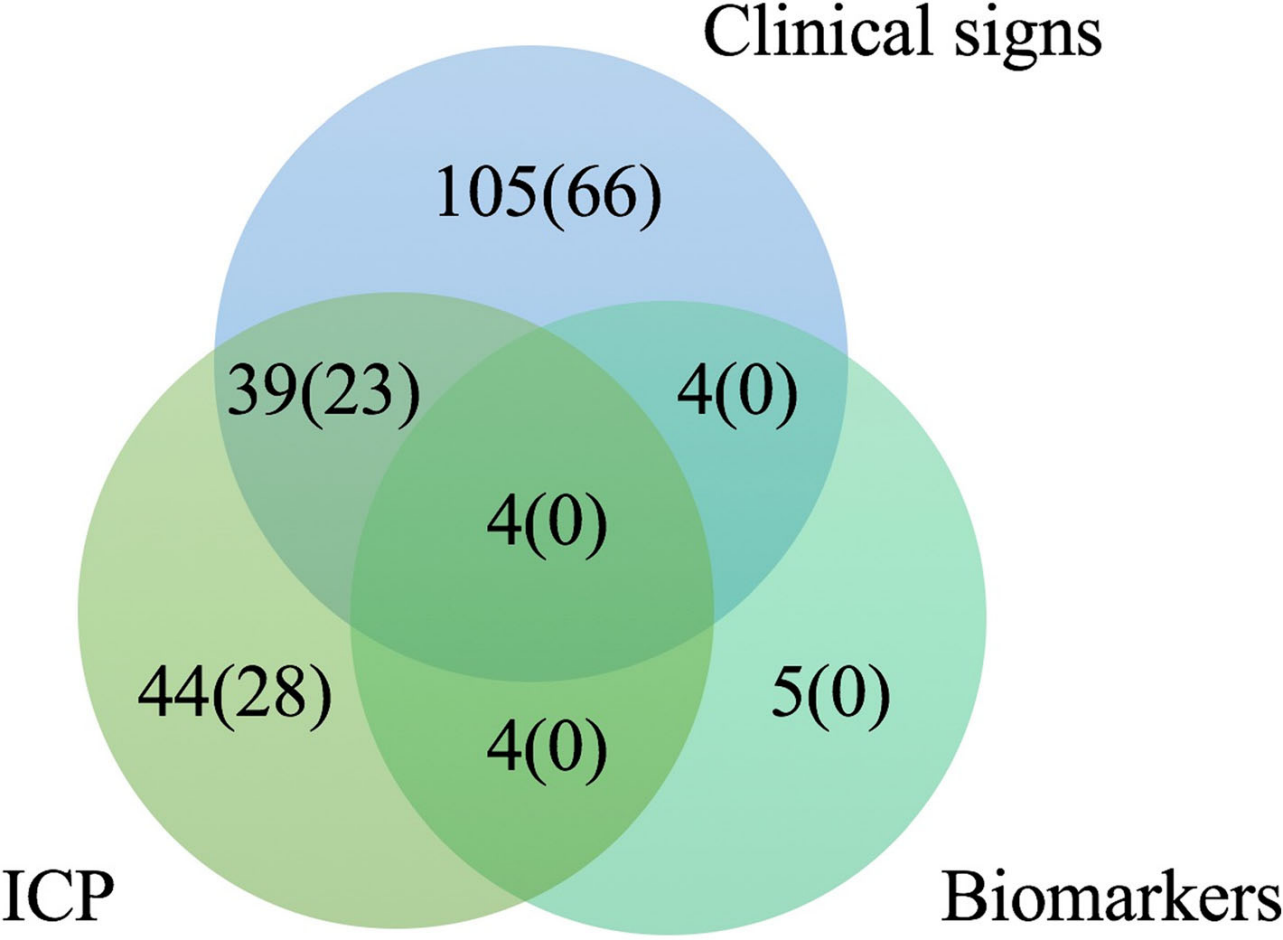
The use of preoperative findings in the decision-making process before a fasciotomy. Values in brackets indicate numbers for fracture patients. ICP (intracompartmental pressure)

The decision to perform fasciotomy was based on clinical findings in 105 cases, intracompartmental pressure measurements in 44, and markers of muscle damage in 5. The combination of clinical findings, pressure measurements, and biomarkers was used in four patients (Fig. 2). In 13 patients with fractures the fasciotomy was performed prophylactically as a preventive measure during a surgical procedure for other indications.

P-myoglobin was analyzed preoperatively in 24 cases (20%), of which 12 were patients with fractures; p-creatine phosphokinase was analyzed preoperatively in 10 patients (8%), of which 1 had a fracture. We defined preoperatively as < 24 h prior to fasciotomy. Reasons why these biomarkers were analyzed could not be determined from the medical notes.

### Fractures compared with non-fractures

The decision to perform fasciotomy was more seldom based on pain in patients with fractures (53 of 79) compared with patients without associated fractures (37 of 42, *p* < 0.05). P-myoglobin was used in the decision making in four patients and p-creatine phosphokinase in one, all without fractures.

**Fig. 3:**
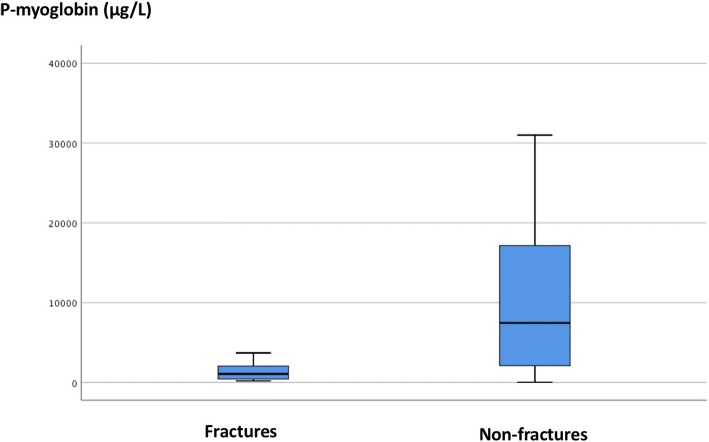
Preoperative median p-myoglobin levels in fracture patients compared with non-fracture patients. Box: 25–75%. Whisker: non-outlier range

Preoperative levels of p-myoglobin were lower in patients with fractures (median 1065 μg/L, range, 200–3700 μg/L) compared with those without fractures (median 7450 μg/L, range 29–31,000 μg/L), *p* < 0.05 (Fig. 3). Median preoperative levels of p-creatine phosphokinase were 72 μkat/L (range 0.87–2300 μkat/L) in patients without fractures. The single preoperative value in patients with fractures was 18 μkat/L.

**Fig. 4:**
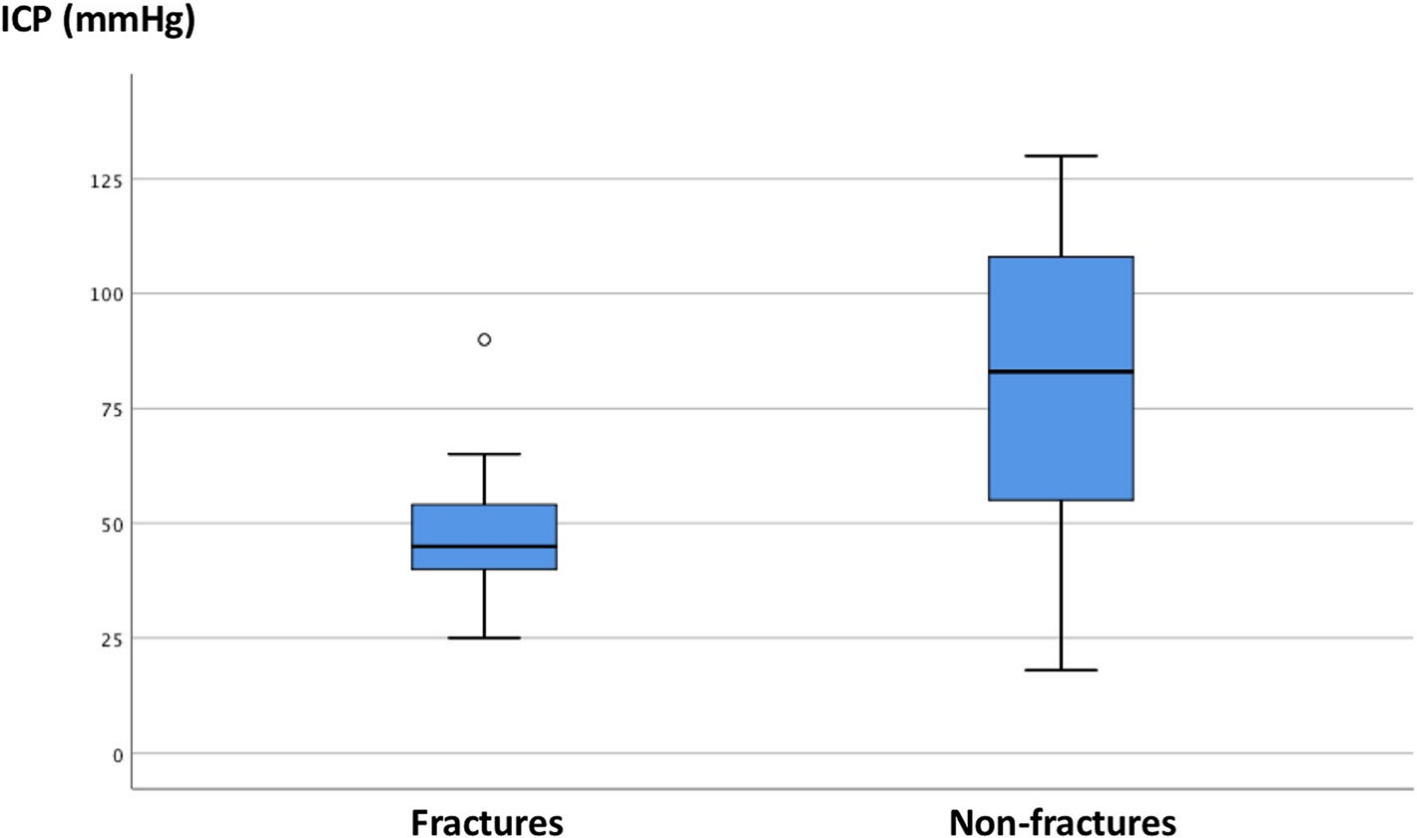
Preoperative median ICP (intracompartmental pressure) values in fracture patients compared with non-fracture patients. Box: 25–75%. Whisker: non-outlier range. Outliers: distance of 1.5 x box height from box end

Intracompartmental pressure (compartment with the highest pressure) was lower in patients with fractures (median 45 mmHg, range 25–90 mmHg) compared with those without fractures (median 83 mmHg, range 18–130 mmHg), *p* < 0.05 (Fig. 4).

Postoperatively, p-myoglobin was analyzed in 41 cases (34%) and p-creatine phosphokinase in 3 (2%). Postoperative levels of p-myoglobin continued to be lower in patients with fractures (median 899 μg/L, range 20–7700 μg/L) compared with those without fractures (median 7000 μg/L, range 110–90,000 μg/L), *p* < 0.05.

We found similar rates in perioperative signs of deteriorated muscle viability and methods of wound closure in both groups (Table 2).

**Table 2:** Perioperative findings

	Fractures (*n* = 79) numbers (%)	Non-Fractures (*n* = 42) numbers (%)
Deteriorated muscle viability, > 3 of 4 Cs	10 (13)	3 (7)
Color	15 (19)	14 (33)
Consistency	36 (46)	11 (26)
Contractility	19 (24)	11 (26)
Capacity to bleed	13 (17)	3 (7)
Delayed closure	75 (95)	31 (74)
Split skin graft	6 (8)	1 (2)
Flap	1 (1)	0 (0)
Amputation	0 (0)	2 (5)

Perioperative findings of deteriorated muscle viability as stated in the medical notes (findings were not documented in roughly 30% of the cases), methods of skin closure after the fasciotomy and amputations

## Discussion

We observed unexpected differences in the use of clinical findings (pain) and biomarkers between patients with acute compartment syndrome and associated tibial fractures and those without fractures. Even more surprising was the finding of highly pathologic levels of p-myoglobin and intracompartmental pressure in patients without fractures, whereas patients with fasciotomies and associated fractures showed lower levels of biomarkers and intracompartmental pressure.

One possible explanation could be differences in the pathomechanism of muscle damage between the two groups. Nonetheless, the lower levels of both intracompartmental pressure and myoglobin in fracture patients are counterintuitive. One would expect already high levels of myoglobin caused by the soft tissue injury associated with the fracture to increase further with the development of an acute compartment syndrome. The opposite finding in our study might indicate a less severe compromise in perfusion pressure, and concomitantly, less severe ischemic muscle damage in fracture patients. An alternative explanation could be a delayed diagnosis in the non-fracture group. These patients may possibly present to health care with delay and diagnosis could be delayed because of a lower degree of suspicion. A longer period of ischemia could result in higher levels of biomarkers. Yet, another explanation might be differences in the size of the affected muscle and the number of muscle compartments affected. However, associated soft tissue injuries should increase the level of biomarkers in the fracture group.

The low frequency of postoperative analyses of p-myoglobin and p-creatine phosphokinase (roughly 30% of the cases) in our study is striking. Fasciotomy is performed to save muscle and neurovascular tissue from cell damage. Even if emergency fasciotomy is performed after the trauma, it is impossible to identify the exact duration of compromised perfusion and therefore the magnitude of cell damage. Blood samples are necessary to diagnose and treat rhabdomyolysis, which otherwise might lead to acute kidney damage. The risk of kidney damage in acute traumatic compartment syndrome is as high as 40% [15]. Low rates of postoperative analyses similar to ours have been reported by others [4] and could possibly indicate a knowledge gap about rhabdomyolysis associated with acute compartment syndrome among treating surgeons.

Our study has several weaknesses. Our data are based on a retrospective review of patients. The fracture group comprises a homogenous group of patients with traumatic fractures of the tibia, whereas the non-fracture group includes patients with a wide variety of underlying conditions. These differences constitute a potential source of confounding that was not corrected for in this study. All data on clinical findings and decision making are based on a review of medical notes with inherent limitations in accuracy. Data on biomarkers and compartment pressure were available only in some patients and therefore selection bias in our comparison may have occurred. Moreover, it was not possible to determine the underlying reasons for the analysis of biomarkers in the medical notes. Finally, the time point for analysis of biomarkers varied in relation to the fasciotomy. To minimize this impact in our comparison we only used values obtained < 24 h before the fasciotomy. Another limitation is that P-myoglobin levels are affected by a number of factors (e.g., liver and kidney function) that we were not able to control.

Currently, acute compartment syndrome is diagnosed by a combination of physical findings and intracompartmental pressure measurements. Each of these have their inherent drawbacks in making the correct diagnosis [10, 16, 17]. Intracompartmental pressure measurements aim to provide an objective tool to measure local tissue pressure and a threshold when to perform fasciotomy [8]. However, once the fasciotomy is done, we can rarely state with confidence whether the patient had a true compartment syndrome and whether fasciotomy was necessary [11]. In our comparison the differences in p-myoglobin follow similar differences in intracompartmental pressure between the groups, suggesting that p-myoglobin and p-creatine phosphokinase could provide a valuable, yet unexplored, diagnostic tool. In one previous study median levels of creatine phosphokinase of ≥4000 U/L [12] and 19,000 U/L [18] were associated with acute compartment syndrome related to other reasons than fractures. For myoglobin, median levels of 1248 μg/L were found in patients with acute compartment syndrome associated with gynecological surgery, whereas median levels in patients without compartment syndrome were much lower (45 μg/L) [18]. In patients with acute femoral embolism treated with embolectomy serum myoglobin was one of several biochemical parameters with a predictive value for the development of acute compartment syndrome during reperfusion [19, 20]. In the context of acute compartment syndrome and tibial fractures no such work has been done.

Our study indicates two things: (1) differences in the mechanism of acute compartment syndrome in fractured and non-fractured patients are reflected in differences of biomarkers and intracompartmental pressure and (2) biomarkers of muscle cell necrosis could possibly play a more important role in the diagnosis of acute compartment syndrome than previously thought.

## References

[1] Matsen FA 3rd, Winquist RA, Krugmire RBJ (1980). Diagnosis and management of compartmental syndromes. J Bone Joint Surg Am.

[2] O'Toole RV, Whitney A, Merchant N, Hui E, Higgins J, Kim TT (2009). Variation in diagnosis of compartment syndrome by surgeons treating tibial shaft fractures. J Trauma.

[3] Dover M, Memon AR, Marafi H, Kelly G, Quinlan JF (2012). Factors associated with persistent sequelae after fasciotomy for acute compartment syndrome. J Orthop Surg.

[4] Lollo L, Grabinsky A (2016). Clinical and functional outcomes of acute lower extremity compartment syndrome at a major trauma hospital. Int J Crit Illn Inj Sci.

[5] Schmidt AH (2011). The impact of compartment syndrome on hospital length of stay and charges among adult patients admitted with a fracture of the tibia. J Orthop Trauma.

[6] Blair JA, Stoops TK, Doarn MC, Kemper D, Erdogan M, Griffing R (2016). Infection and nonunion after fasciotomy for compartment syndrome associated with tibia fractures: a matched cohort comparison. J Orthop Trauma.

[7] Whitesides TE, Haney TC, Morimoto K, Harada H (1975). Tissue pressure measurements as a determinant for the need of fasciotomy. Clin Orthop Relat Res.

[8] McQueen MM, Court-Brown CM (1996). Compartment monitoring in tibial fractures. The pressure threshold for decompression. J Bone Joint Surg Br.

[9] McQueen MM, Duckworth AD, Aitken SA, Court-Brown CM (2013). The estimated sensitivity and specificity of compartment pressure monitoring for acute compartment syndrome. J Bone Joint Surg Am.

[10] Schmidt AH (2013). Continuous compartment pressure monitoring-better than clinical assessment?. J Bone Joint Surg Am.

[11] Prayson MJ, Chen JL, Hampers D, Vogt M, Fenwick J, Meredick R (2006). Baseline compartment pressure measurements in isolated lower extremity fractures without clinical compartment syndrome. J Trauma.

[12] Valdez C, Schroeder E, Amdur R, Pascual J, Sarani B (2013). Serum creatine kinase levels are associated with extremity compartment syndrome. J Trauma Acute Care Surg.

[13] Shadgan B, Menon M, O'Brien PJ, Reid WD (2008). Diagnostic techniques in acute compartment syndrome of the leg. J Orthop Trauma.

[14] Schatzker J, McBroom R, Bruce D. The tibial plateau fracture. The Toronto experience 1968--1975. Clin Orthop Relat Res. 1979;(138):94–104. 445923

[15] Tsai WH, Huang ST, Liu WC, Chen LW, Yang KC, Hsu KC (2015). High risk of rhabdomyolysis and acute kidney injury after traumatic limb compartment syndrome. Ann Plast Surg.

[16] Shuler FD, Dietz MJ (2010). Physicians' ability to manually detect isolated elevations in leg intracompartmental pressure. J Bone Joint Surg Am.

[17] Heckman MM, Whitesides TEJ, Grewe SR, Rooks MD (1994). Compartment pressure in association with closed tibial fractures. The relationship between tissue pressure, compartment, and the distance from the site of the fracture. J Bone Joint Surg Am.

[18] Hefler-Frischmuth K, Lafleur J, Brunnmayr-Petkin G, Roithmeier F, Unterrichter V, Hefler L (2017). Compartment syndrome after gynecologic laparoscopy: systematic review of the literature and establishment of normal values for postoperative serum creatine kinase and myoglobin levels. Arch Gynecol Obstet.

[19] Mitas P, Vejrazka M, Hruby J, Spunda R, Pecha O, Lindner J (2014). Prediction of compartment syndrome based on analysis of biochemical parameters. Ann Vasc Surg.

[20] Adiseshiah M, Round JM, Jones DA (1992). Reperfusion injury in skeletal muscle: a prospective study in patients with acute limb ischaemia and claudicants treated by revascularization. Br J Surg.

